# Trends in Caregiving, Work, and Caregiving-Related Work Consequences for Caregivers of Older Adults

**DOI:** 10.1093/geroni/igaf122.923

**Published:** 2025-12-31

**Authors:** Sarah Patterson, Vicki Freedman, Jennifer Cornman, Jennifer Wolff

**Affiliations:** University of Michigan, Ann Arbor, Michigan, United States; University of Michigan, Ann Arbor, Michigan, United States; Columbus, Ohio, United States; Johns Hopkins University - School of Public Health, Baltimore, Maryland, United States

## Abstract

Both men and women continue to have high rates of employment at the same time that more family and unpaid caregivers are providing help to an older adult. We use the National Study of Caregiving (NSOC) 2011 - 2023 to investigate trends among working caregivers to older adults. Findings reveal that the number of working caregivers to older adults grew from 7.8 million in 2011 to 9.6 million in 2023. Women comprised more than 70% of working caregivers in both years. The percentage of working caregivers assisting older adults with dementia decreased for both men and women, from about half in 2011 to slightly more than a third in 2023. Among working caregivers, women were more likely than men to experience distractions at work related to caregiving, or “presenteeism” in 2011 (7.5. vs. 3.0; p<.01); but men experienced a significant increase in presenteeism from 2011 to 2023 (3.0 to 7.1; p<.05), which closed the gap (7.1 men, 9.1 women, n.s.). Similar patterns were found for work productivity loss (a measure combining absenteeism and presenteeism). Trends in the percentage providing specific types of care converged for working men and women for personal care, chores, ordering medications, and health-care related activities (with men’s provision of these types of care no longer significantly lower than women’s provision). Results have implications for policies and programs aimed at supporting working caregivers of older adults.

